# Potentials and Challenges of Former Food Products (Food Leftover) as Alternative Feed Ingredients

**DOI:** 10.3390/ani10010125

**Published:** 2020-01-13

**Authors:** Alice Luciano, Marco Tretola, Matteo Ottoboni, Antonella Baldi, Donata Cattaneo, Luciano Pinotti

**Affiliations:** 1 Department of Health, Animal Science and Food Safety, VESPA, University of Milan, 20134 Milano, Italy; alice.luciano@unimi.it (A.L.); matteo.ottoboni@unimi.it (M.O.); antonella.baldi@unimi.it (A.B.); donata.cattaneo@unimi.it (D.C.); 2 Agroscope, Institute for Livestock Sciences, 1725 Posieux, Switzerland; marco.tretola@agroscope.admin.ch

**Keywords:** former foodstuff, ex-food, alternative feed ingredients, predicted glycaemic index, gut microbiota, feed safety, pigs

## Abstract

**Simple Summary:**

This review focuses on the use of ex-foods, an alternative feed ingredient in farm animal diets, composed by processed and ready-to-eat food products no longer suitable for human consumption. Such foods, which are also called former food products, are usually buried in landfill sites, despite their high potential of being used as sustainable feed ingredients. In order to obtain proper balanced diets by using these alternative feed ingredients, several aspects have to be considered. In this respect, this paper aims to address the state of the art about food leftovers used in animal nutrition in general and in pig diets specifically.

**Abstract:**

Former food products (FFPs) are foodstuffs that, even though they are nutritious and safe, have lost their value on the human consumption market for different reasons, such as production errors leading to broken or intermediate foodstuffs, surpluses caused by logistical challenges of daily delivery, or any other reason. The nutritional features of FFPs include carbohydrates, free sugars, and possibly also fats. FFPs tend to have been processed through various technological and heat treatments that impact the nutrients and the kinetics of digestion, as well as animal response and, particularly, gastro-intestinal health. This review integrates some of the most recently published works about the chemical composition, nutritional value, digestibility and glycaemic index of ex-foods. In addition, a view on the relationship between the use of FFPs and safety issues and their effects on pigs’ intestinal microbiota are also given.

## 1. Introduction

Nowadays agriculture, and even more so livestock production, are faced with a wide range of complex challenges. From the perspective of sustainability, livestock production has received considerable attention in recent years over the extent to which animal feed production competes for land and other resources with the production of human food. Livestock consumes a third of all cereals produced and uses about 40% of global arable land. In fact, farm animals occupy two billion ha of grasslands, of which about 700 million ha could be used to grow crops. From another perspective, 86% of the plant material fed to livestock would be inedible by humans directly, but it is instead converted into valuable food for human consumption (e.g., milk, meat), thus contributing greatly to food and nutrition security [1]. In general, it has been estimated by the Food and Agriculture Organization of the United Nations (FAO) that about 3 kg of human-edible material, mostly grains, are needed to produce 1 kg of meat. These global figures, however, have to be considered with caution, since wide differences across species and production systems exist. While ruminants consume more dry matter per kg of protein produced compared to pigs or poultry, they require less human-edible protein, since they can rely more on grass and forages. Pigs and poultry consume less feed to produce the same amount of protein, but a far higher proportion of what they do consume could be eaten directly by humans [1]. In livestock production systems, the cost of animal feed represents up to 85% of the farm gate value of several animal products [2]. In light of this, proper feeding and nutrition strategies are becoming increasingly important as livestock systems strive to become more efficient. In this scenario, the use of alternative feed ingredients in farm animal’s diet can be an fascinating option from several standpoints, and ex-food recycling is an interesting model. By definition, “Ex-food” or “Former foodstuffs” (FFPs) means foodstuffs which were manufactured for human consumption in full compliance with the EU food law, but which are no longer intended for human consumption for practical or logistical reasons and which do not present any health risks when used as feed [3]. It has been estimated [4] that 3–3.5 Mt of FFPs are processed in the EU. Ex-foods are already used in animal nutrition (they are in the EU’s feed catalogues), but to a limited extent (3.3%) compared to the total food waste. The potential of these products has not been fully exploited yet as feed ingredients. The target species are omnivores, such as pigs and poultry, even though their use in ruminants cannot be excluded.

Examples of FFPs include various leftovers from the food industry: pasta, bread, cereals, savoury snacks, biscuits, sweets and chocolate bars. Such foods are rich in sugar, starch, oil or fat, thus giving them a high energy content [5,6,7]. 

Livestock systems today and in the future have to take into account not only economic development and feed security and safety, but also politically-sensitive issues such as animal welfare and environmental sustainability. Sustainable feed/food security is thus in need of innovation [8] and the conversion of industrial food losses into ingredients for animal feed maintains such losses in the food chain and should thus be implemented on a global basis [9]. In this respect, this paper aims to address the state of the art about the use of ex-food in animal nutrition, with special emphasis on their nutritional properties and safety issue. 

## 2. Former Food Products: Nutrient Content and Dietetics

**Figure 1:**
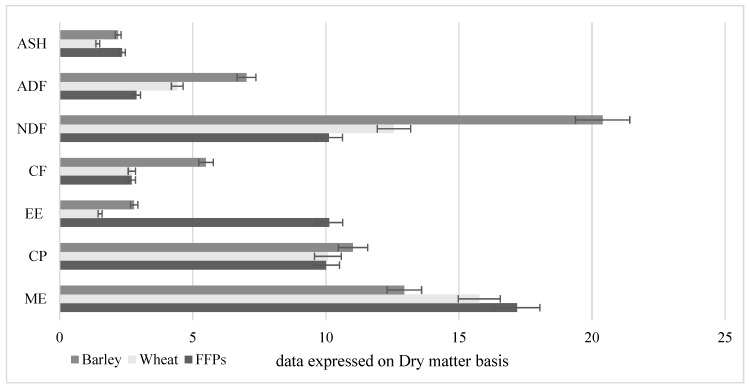
Nutrients—ash, acid detergent fibre (ADF), neutral detergent fiber (NDF), crude fiber (CF), fat (ether extract—EE), crude protein (CP); all expressed as % and energy content (ME, MJ kg^−1^) of FFPs [5,7].

Former food products or ex-food are defined in the Regulation (EC) No 68/2013 as “foodstuff other than catering reflux, which were manufactured in full compliance with EU food law but are no longer intended for human consumption for practical and logistical reasons or due to problems in manufacturing or packaging which are unlikely to cause any health risks when used as feed”. These materials are dried and sorted, unpacked, ground and sieved to create new feed ingredients, which can be use as substitute of existing raw materials in various farm animal compound feeds [5,6,7]. Ex-food ingredients can be divided in two main categories: leftovers of the food industry mainly composed by bakery products (i.e., bread, pasta etc.) and leftovers of the food industry principally composed by confectionery products (e.g., chocolates, biscuits etc.). Bread and salty cakes/snacks, due to the long baking process, represent a macerated and easy to digest source of energy with high starch contents. Confectionary products that consist, for example, of chocolate, dry cakes and biscuits, waffles, and muesli products can be considered supplemental feed, available all year round and rich in simple sugars, fat and energy. In light of these features the main animal targets for FFPs are young animals, e.g., piglets, chicks and calves, due to the high amount of digestible carbohydrates, like cooked starch. Indeed, cooked starch food represent a rich source of rapidly digestible starch and rapidly available glucose, features that can strongly affect productive performances (such as feed intake) and nutrient digestibility. Moreover, thanks to the ingredient used in their preparation (e.g., butter sweet and chocolates), FFPs are often rich in fats [5,6,7]. These properties have been studied by Giromini et al. [5], who reported that bakery and confectionary ex-food- processed for pig nutrition- have a nutrient content similar to wheat and barley grains, although with a higher energy content (Figure 1). Mean FFP’s metabolizable energy (ME) content was 16.95 MJ kg^−1^ with fats and starch being the main contributors. FFPs have a lipid content of around 10%–12%, which is three to six times than reported for wheat and corn. The starch content in FFPs can be up to 50%–60% on dry matter basis (DM). Former food products have also shown high digestibility values, which in the above mentioned study [5] ranged from 79% up to 93% DM, depending on how the ex-food was mixed and prepared. The mean protein content in the FFPs was around 10.0%, consequently FFPs should not be considered as a valuable source of protein. These features are summarised in Figure 1. 

The free/simple sugar content of FFPs is another key quality aspect with a positive impact on the digestion kinetics of carbohydrates and which also boosts the glycaemic index (GI). In human nutrition, the GI is used to classify starchy foods based on their post-prandial glucose release into the bloodstream [10]. In terms of livestock, the glycaemic index was initially used in equine (racing horses) nutrition in relation to disorders associated with the carbohydrate metabolism [11]. It was then introduced for pig nutrition by Menoyo et al. [12] in order to classify cereals. Cereals and food preparation with a high GI tend to promote insulin production with a consequent increased feed consumption. 

**Figure 2:**
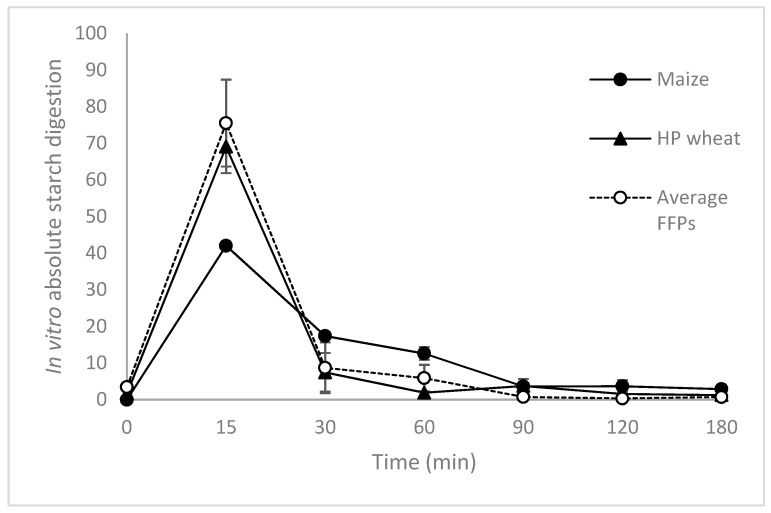
Absolute in vitro total carbohydrate digestion (as a fraction of total carbohydrates/min) of cereal grains (unprocessed maize and heat processed wheat) and former food products (FFPs). Adapted from [13].

As previously reported, FFPs are produced starting from food leftover that have been cooked and/or heat-treated during their production process in the food industry [5,6,7]. As a result, these materials are characterized by a higher digestibility compared to the cereal grains commonly used in farm animal diets in general, and pig nutrition particularly [7]. Processing techniques (e.g., thermal processing, extrusion cooking etc.) are able to affect both digestibility and absorption of digested carbohydrates [13], which in turn have a major impact on the glycaemic index [10]. These dietetics features have been investigated by Ottoboni et al. [13], who measured hydrolysis index (HI), predicted glycaemic index (pGI), and the time trend in carbohydrate digestion (k), in FFPs in comparison with common cereals. Results obtained indicated that all parameters related to carbohydrate digestion (i.e., HI, pGI and k) were always higher in ex-food compared to conventional cereals feed ingredients such as unprocessed corn [13] (Figure 2). However, it is known that other constituents of the food matrix, such as proteins, lipids and fibres, play a significant role during processing which affects the physico-chemical characteristics of digesta and the final digestibility of starch [13]. In this respect, a further step in Ottoboni’s study was to evaluate FFPs, not only as single ingredient but also when they were included in a pig formula. Data obtained on two post-weaning piglet complete diets (a cereal based vs. a FFPs diets) clearly indicated that the inclusion of FFPs (up to 30%) as a substitute for cereals (corn, wheat, de hulled barley) has produced a big impact on in vitro starch hydrolysis kinetics and digestibility. Substitution of common cereal with FFPs in piglet diets has improved starch susceptibility to enzymic digestion, thus probably optimizing their nutritional/dietetic quality [10,11,12,13]. This implies a functional evaluation with special emphasis on FFPs’ impact on animal welfare in general and the gastro-intestinal tract (i.e., gut health), in particular [13,14]. 

**Figure 3:**
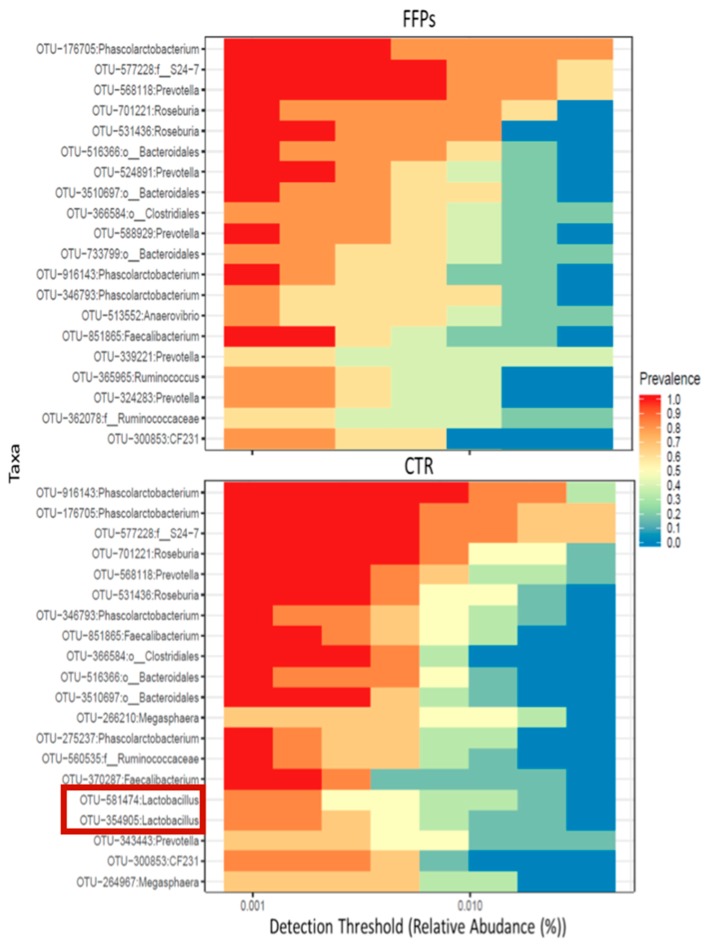
Large intestine microbiota with specific keystone taxa detected at the end of the experiment (D16) in piglets receiving (FFPs) or not (CTR) former foods products (30% inclusion) in their diet. Red box evidences lactobacillus strains; adapted from Tretola et al. [19].

The FFPs, however, might contain more than 20% of simple sugars, that can affect not only the gut transit, but also its health and ecology [15,16]. Understanding what a healthy microbiota looks like and how FFPs can influence the composition of the gut microbial population, improving eubiosis and/or reducing dysbiosis, provides fundamental information to efficiently reconvert FFPs into value added products for animal nutrition. Furthermore, the diet-driven different modulation of the gut microbiota can affect the local and systemic setting of immunity [15,16,17]. This assessment in general requires the combination of several different approaches that include in vivo studies. In this direction, recent studies have been conducted in order to investigate the effect of FFPs on growth performance [18] and gut microbiota in weaning pigs [19]. In these studies, the authors evaluated the effects of substituting 30% conventional cereals for 30% FFPs in post-weaning piglet’s diets [18,19]. The results obtained indicated that both in vitro and in vivo digestibility values were higher for FFPs diets compared to the control ones. Both average daily gain and feed intake were not affected by dietary treatment. Conversely, piglets on the FFPs diet showed a lower feed conversion rate. Therefore, it can be suggested that inclusion of FFPs -up to a level of 30% as cereal substitute- in post-weaning diets, has no detrimental effects on pig growth performance [18]. Moreover, large intestine microbial taxa composition has shown no major modifications [19]. Specifically, FFPs diet decreased the microbiota diversity/richness and evenness in the large intestine while minor differences have been observed in taxa composition. The main changes in the FFP group over time affected the *Bacteroidetes*, which increased during the first period (27% and 48% in day 0 and day 8, respectively), and decreased again to the original values (29%) in the last sampling day. Thus, FFPs led to a qualitative modification in the gut microbial community over time. Similarly, at the end of the trial FFP diet increased the amount of the *Proteobacteria* phylum and decreased the abundance of *Lactobacillus* genus, compared to the control diet (Figure 3). Even though no gastrointestinal disorders have been recorded during the trial, these differences observed at the end of the study should be considered with caution in terms of gut health. The phylum of *Proteobacteria*, in fact, includes several opportunistic pathogens often associated with gastrointestinal disorders both in animals and humans. In contrast, a decreased abundance of the bacteria belonging to the *Lactobacillus* genus, could result in a reduction of health-promoting probiotics [19]. However, since the core microbiota composition was slightly affected, the potential impacts of FFPs on microbiota require further investigation with a wider panel of conditions and exposure time.

Nutritional properties of FFPs, however, are not the only plus of these materials. The use of FFPs in farm animal diets has also a big potential in terms of feed processing/manufacturing and technological quality. They are indeed energy dense ingredients often characterized by a valuable fat concentration. This can be considered a technological benefit since lipids are already embedded in the feed matrix, which means that they can be easily manipulated and processed during feed production, since there is no need for their addition. This technological feature not only facilitates feed production, but also increases the energy density of the diets. These characteristics are even more important in modern lean pig strains (average daily gain > 1kg) which have high energy requirements and impose a need for nutritious and energy dense ingredients. 

In summary, from the perspective of the circular economy, reprocessing FFP biomass is particularly attractive and sustainable, limiting food losses and the competition for human edible cereals.

## 3. Safety Concerns in Former Foods Products

Recycling ex-food in the feed sector involves a combination of different processes, which are related to the type of food. These processes include collection, unpacking, mixing, grinding and drying, that impact both quality and safety. In terms of safety, both microbiological load and packaging remnants are the main issues for the current regulations on feed standards [6,20,21,22,23]. 

With regards to microbiological quality, Tretola and co-workers [6] investigated the different FFPs. In this study the first indicator used to evaluate the general hygienic condition of feedstuff was the total viable count (TVC). The recorded values for TVC were, for all tested FFPs samples, below 5 log CFU g^−1^. None of the samples exceeded the microbial loads of 6 log CFU g^−1^, which is generally recognized in food as the threshold limit above which spoilage could occur [6]. The limited microbiological load was also confirmed when different microorganisms were considered. The mean count of *Enterobacteriaceae* was also limited, confirming the low level of bacterial contamination. Both the *E. coli* and *Staphylococci* count were below the detection limit or extremely low (≤2 log CFU g^−1^), respectively. The same was for *B. cereus* and its spores, which are considered indicators of poor processing, poor quality of raw materials, or poor temperature control. In tests of FFPs, theses strains never exceed the level of 5 log CFU g^−1^, known as the starting concentration from which toxin production may occur. Likewise, *Clostridia* were found to be countable just in a limited number of FFPs samples and in very low loads (1–1.7 log CFU g^−1^); levels around 1 log CFU g^−1^ are considered satisfactory and commonly levels below 4 log CFU g^−1^ are considered not of particular apprehension. Yeasts and moulds, which are among the most critical organisms for this type of feedstuff, were present in very small quantities, confirming again stability of these materials [6]. However, the major hazard for the microbial contamination of animal feed is *Salmonella spp*. Of note, in all FFPs tested in the study [6], *Salmonella spp.* was never detected, matching the standard established by the main health authorities for the animal feed sector [3,6,21]. These results, however, were expected, as the tested FFPs were dry and cooked at high temperature during the production process, that probably affected their microbiological stability.

A further safety issue in FFPs use and application in animal nutrition is related to the presence of packaging remnants. Packaging materials are generally not accepted as a feed ingredient in accordance with the feed standard regulations [6]. In terms of packaging remnants, a useful example is represented by bakery co-by-products such as bread, biscuits, waffles, and breakfast cereals whose packaging must ensure the maintenance of quality during transport and storage. Food packaging vary widely based on the materials used and on how the food has been processed [6,20,22,23,24]. 

Plastic is the packaging material most commonly used in food industries. To a lesser extent, aluminium, resin, and pressed paperboard are used [22,24]. The main types of materials used are polyolefin such as polypropylene and polyethylene. Polypropylene can resist temperatures of up to 220 to 240 °C and tends to be made in black or clear, very rigid, crack-resistant. Polyethylene has an average melting point of 120 °C. Five other commonly used polyolefins are: (i) polyethylene terephthalate and its copolymers, which melt before 140 °C and are found in different colours; (ii) polystyrene, which has a moderate resistance to temperature and is found in a variety of colours; (iii) pressed paperboard, which resists in an oven for an hour at temperatures of up to 200 °C and which is manufactured in a variety of colours and patterns; iv) rigid polyvinylchloride (PVC, regenerated cellulose (RC)); and finally (v) aluminium foil (silver or coated in colours and can withstand very high temperatures) [22,24]. However, in spite of this variability of packaging materials, data available in the literature [6,20,22,23] indicate that packaging remnants in FFPs are usually negligible (<0.10 g/100 g). 

## 4. Conclusions

Mitigating environmental impact is crucial to sustainable production in the livestock sector. This can be achieved by reducing food waste through recycling, and especially by enhancing the management of FPPs, with the added benefit of being an economic resource. As with other alternative/innovative feed ingredients [25,26,27,28], exploiting FFPs in feed production fully meets the requirements of the circular economy. From the food supply industry, there are always unintentional and unavoidable food losses, which preclude foodstuffs from reaching the human food market. In this context, FFPs are seen as a potential resource rather than a waste product sent to landfill or otherwise disposed of in the natural environment. Their potential seems higher for omnivorous farm species (e.g., pigs and poultry) even though some studies, mainly on bakery products, have opened new frontiers in ruminants’ nutrition [29,30]. This will therefore save on costs and reduce the impact of livestock production on the environment.
